# Comparison of the Association of Sac Growth and Coil Compaction with Recurrence in Coil Embolized Cerebral Aneurysms

**DOI:** 10.1371/journal.pone.0123017

**Published:** 2015-04-20

**Authors:** Anna L. Hoppe, Madhavan L. Raghavan, David M. Hasan

**Affiliations:** 1 Department of Biomedical Engineering, University of Iowa, Iowa City, Iowa, United States of America; 2 Department of Neurosurgery, University of Iowa Carver College of Medicine, Iowa City, Iowa, United States of America; University of Washington, UNITED STATES

## Abstract

**Background and Purpose:**

In recurrent cerebral aneurysms treated by coil embolization, coil compaction is regarded as the presumptive mechanism. We test the hypothesis that aneurysm growth is the primary recurrence mechanism. We also test the hypothesis that the coil mass will translate a measurable extent when recurrence occurs.

**Methods:**

An objective, quantitative image analysis protocol was developed to determine the volumes of aneurysms and coil masses during initial and follow-up visits from 3D rotational angiograms. The population consisted of 15 recurrence and 12 non-recurrence control aneurysms initially completely coiled at a single center. An investigator sensitivity study was performed to assess the objectivity of the methods. Paired Wilcoxon tests (p<0.05, one-tailed) were performed to assess for aneurysm and coil growth. The translation of the coil mass center at follow-up was computed. A Mann Whitney U-Test (p<0.05, one-tailed) was used to compare translation of coil mass centers between recurrence and control subjects.

**Results:**

Image analysis protocol was found to be insensitive to the investigator. Aneurysm growth was evident in the recurrence cohort (p=0.003) but not the control (p=0.136). There was no evidence of coil compaction in either the recurrence or control cohorts (recurrence: p=0.339; control: p=0.429). The translation of the coil mass centers was found to be significantly larger in the recurrence cohort than the control cohort (p=0.047).

**Conclusion:**

Aneurysm sac growth, not coil compaction, was the primary mechanism of recurrence following successful coil embolization. The coil mass likely translates to a measurable extent when recurrence occurs and has the potential to serve as a non-angiographic recurrence marker.

## Introduction

Identifying the mechanism underlying cerebral aneurysm recurrence will improve patient selection for coil embolization treatment, as well as impact coil device design.[1–3] Raymond et al.[4] and Murayama et al.[5] reported recurrence rates of 33% and 21% over longitudinal follow-up. Compaction of the coil mass is a plausible mechanism but there is not sufficient quantitative evidence to justify it being the presumptive mechanism, as the supporting evidence usually collected are visual inspections of 2D angiograms.[1–3] We submit that differences in magnification and patient orientation between x-ray scans can make it difficult for neuroradiologists to correctly diagnose coil compaction; such differences can even be misleading. Growth of the aneurysm sac is an alternative recurrence mechanism, but reports about it in the literature are scarce.[1, 6–8] Therefore the primary goals of this study were to perform an assessment of the relative incidences of sac growth and coil compaction in a study population larger than that used in our earlier report [9], but more importantly, to develop an objective image analysis protocol and demonstrate its fidelity by including non-recurrence controls. Specifically we reported in an earlier study that sac growth may be equally as or more important than coil compaction as a mechanism for recurrence.[9] A similar study conducted even earlier by De Craene et. al.[10] with four patients also reported that all showed longitudinal sac growth while coil compaction was only found in one case.[10] It is remarkable that two independent studies, explicitly comparing sac growth and coil compaction as etiologies for recurrence found that, contrary to conventional thinking, sac growth may be as often or more often associated with it. Some caution is however warranted in drawing any conclusive claims from these reports because in addition to small study population size, they suffer from one key limitation: neither demonstrated the fidelity of the image processing methods by using non-recurrence controls to establish specificity.

We also sought to test the hypothesis that there is measurably greater translation in the coil masses of aneurysms that present with recurrence compared to control aneurysms. This hypothesis is based on the following logic: if recanalization at the aneurysm neck is indeed due to sac growth causing the coil mass to move deeper into the sac, than coil mass movement (or translation) should be greater in aneurysms that recur than in those that do not. This is an important hypothesis to test because if translation changes in a coil mass has sufficient sensitivity and specificity as a marker for recurrence then patients may be screened for recurrence using cheap, convenient and non-invasive non-angiographic scans rather than angiograms. While the idea of using non-angiographic scans to identify coil mass translation as a proxy for recurrence is not new, Hwang et al.[11] proposed it in 2000, we are the first group to rigorously compute coil mass translation in a population of both recurrence and control aneurysms.

## Materials and Methods

### Study Population

Between July 2009 and September 2012, 412 intracranial aneurysm patients were treated with coil embolization at the University of Iowa Hospitals and Clinics (UIHC). Of these 412 patients, 15 patients with 15 aneurysms were found by the attending neuroradiologist to have recurrence sufficient enough to necessitate re-treatment when they presented at follow-up (2–24 months between procedures; mean: 9.5 months). These 15 aneurysms (recurrence cohort) were the only aneurysms that presented with recurrence at the UIHC between July 2009 and September 2012 with follow-up three-dimensional rotational angiography (3DRA) scans. Ten control patients with 12 aneurysms were chosen consecutively from the rest, beginning at the start of the study (control cohort). These control aneurysms were completely coiled initially and remained stable over the follow-up period (5–23 months between procedures; mean: 10.2 months). It should be noted that all aneurysms in this study were coiled by a single neuroradiologist to the point that there was no residual in the neck or dome. Institutional review board approval (No. 201210828) and patient informed consent was obtained prior to beginning the study.

### Image Processing Protocol

To independently assess coil mass compaction and aneurysm growth, 3DRA scans were first collected retrospectively for the two groups. The 3DRA image acquisition was done using Siemens’ Syngo InPace 3D Workstation (in-plane resolution: 0.22 mm^2^, out-of-plane resolution: 0.22 mm). The 3DRA scans obtained for the recurrence patients were from four time points: pre-first treatment (1-), post-first treatment (1+), pre-second treatment (2-), and post-second treatment (2+). In contrast, the 3DRA scans obtained for the control patients were from three time points: 1-, 1+, and 2- since they do not undergo a second coiling treatment. Three-dimensional models of the aneurysm sac, contiguous vessels, and aneurysm coil mass at each study time point were then created from the 3DRA scans using a dedicated image processing pipeline. This pipeline was built using Vascular Modeling Toolkit (VMTK) 1.0.0 libraries (Antiga and Steinman 2004–2013); additionally, some new VMTK libraries were also developed such that automated image segmentation and automated aneurysm sac isolation could be implemented, for example. It should be noted that the image processing pipeline published by our group in the pilot study[9] used a more manual approach to create the aneurysm and coil mass models; the new image processing pipeline was developed to ensure automatic, objective and an investigator insensitive image analysis.

**Fig 1:**
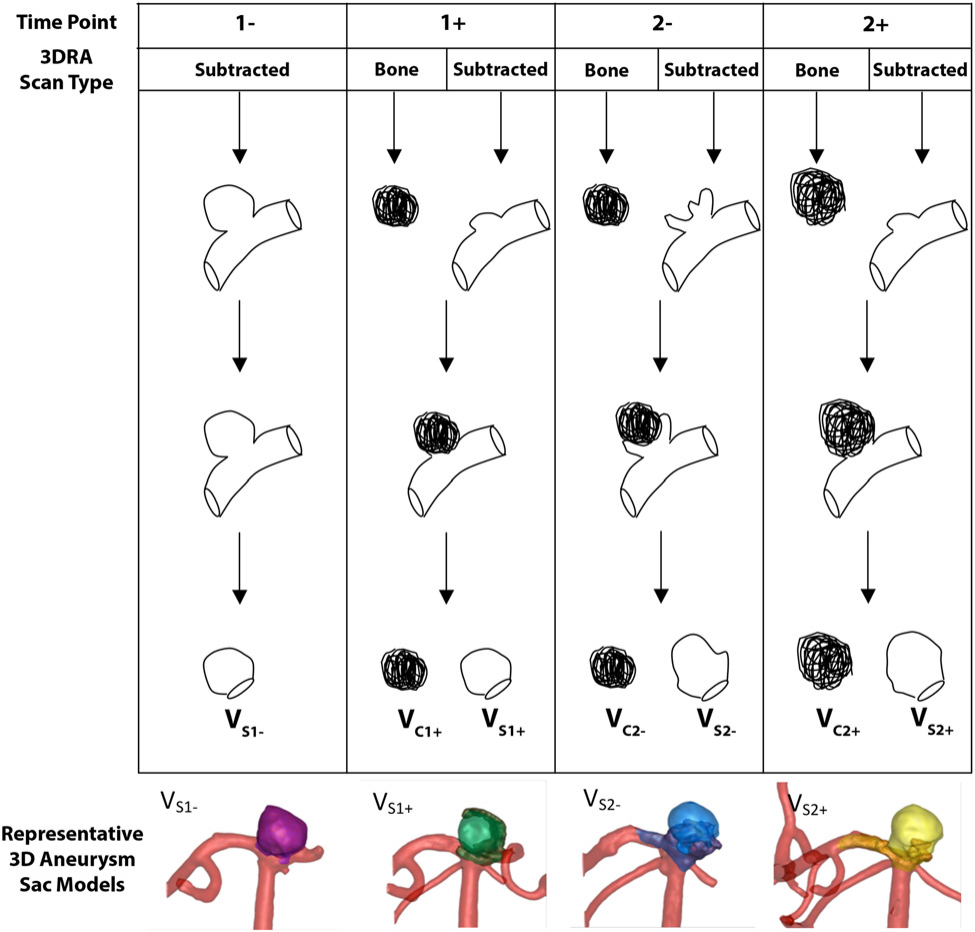
Schematic illustration of the image processing protocol. ‘1- ‘ indicates the pre-first coiling treatment time point; ‘1+’, post-first coiling treatment time point; ‘2-’, pre-second coiling treatment time point; ‘2+’, post-second coiling treatment time point; 3DRA, 3D rotational angiogram. The first column depicts the image processing protocol for the 1- time point, beginning with generation of the aneurysm and vessel model from the subtracted 3DRA scan. The aneurysm sac is then automatically isolated from the vasculature. A representative 1- aneurysm sac model is shown at the bottom of column 1. The adjacent column details the workflow for analyzing the data from the 1+ time point. At this time point the coil mass model is generated from the baseline (or bone) 3DRA scan, while the vessel and residual blood model is generated from the subtracted angiographic scan. The coil mass, vessel and residual blood models are then added together by Boolean union. The aneurysm sac is the combination of the coil mass and any outlying residual blood, of which the aneurysm neck surface is automatically determined. A representative 1+ aneurysm sac model is shown at the bottom of column 2. The remaining two columns outline a similar workflow for analyzing data from the 2- and 2+ time points respectively.

The image processing algorithms implemented to generate the 3D aneurysm and coil mass models differ slightly depending upon whether the 1- or any of the later time point 3DRA scans (1+, 2-, or 2+) are identified as input. This is because the aneurysm sac is directly visible in the 1- scan but not in the 1+, 2-, or 2+ scans. The 1- aneurysm sac models were each generated from their corresponding subtracted 3DRA image volume because this image features only vessel information. To generate the 3D aneurysm and vessel model, an image segmentation containing both the blood flowing through the contiguous vessels and any blood filling the aneurysm sac was first created. To do this a grayscale morphological opening filter was applied to the subtracted image volume in order to rid it of small-scale noise. Next the user manually places a seed point inside the aneurysm sac; this seed point serves only as an initialization point for the segmentation algorithm and its placement does not affect the segmentation boundaries. Otsu’s algorithm[12] was implemented to generate the segmentation. This algorithm works by finding the statistically optimal threshold between foreground and background voxels in an image volume of interest (VOI).[12] The segmentation was then further evolved using the level set algorithm[13] and the associated 3D model was created from it using the marching cubes algorithm.[14] The aneurysm sac was then isolated from the contiguous vasculature automatically, the result of which was a 3D sac model with a non-planar neck surface per the approach proposed by Ford et al.[15] (see Fig 1). This approach is superior to the use of a single cutting plane to isolate the aneurysm because it retains more of the sac surface features. The 3D models of the 1+, 2-, and 2+ coil masses were each created from the baseline (bone scan) 3DRA image volumes. This was done because the baseline image volumes feature only skull and coil information. A combination of erode and dilate filters were first applied to these image volumes in order to rid the geometry of holes internal to the bounding coil wire. If a hole passed through the entire coil, rendering it a non-simply connected geometry, then this hole was retained. This was done because it was unclear how to fill such a hole in the absence of a bounding coil wire. The resulting coil mass region, representing both the coil wires and interstitial thrombi, was collectively referred to as the coil mass. Similar to the aneurysm and vessel segmentations, Otsu’s segmentation algorithm[12] was then implemented to generate the coil mass segmentation. This segmentation was further evolved using the level set algorithm[13] and the associated 3D model of the coil masses at 1+, 2- and 2+ time periods were created using the marching cubes algorithm.[14] Subsequently, the angiographic volumes (contiguous vessels and residual regions, if any) for 1+, 2- and 2+ time periods were segmented as done for the 1- time period. The aneurysm sac models for 1+, 2- and 2+ were then generated by isolating the sac[15] from the Boolean union of the angiographic volume with the coil mass volume (see Fig 1).

The volumes of the following structures were computed from the representative aneurysm and coil mass models: the aneurysm sac volume at the 1- time point (*V*_S1-_), the aneurysm sac volume at the 1+ time point (*V*_S1+_), the aneurysm sac volume at the 2- time point (*V*_S2-_), the aneurysm sac volume at the 2+ time point (*V*_S2+_), the coil mass volume at the 1+ time point (*V*_C1+_), and the coil mass volume at the 2- time point (*V*_C2-_). Volumetric sac growth, *V*_SG_, was computed as the difference in sac volume between follow-up and initial presentations. Using this definition *V*_SG_ > 0 indicates growth of the sac at follow-up. However given the four study time points (1-, 1+, 2-, and 2+) there can be two sac volumes at initial presentation (*V*_S1-_, *V*_S1+_); similarly there can be two sac volumes at follow-up (*V*_S2-_, *V*_S2+_). In theory, these volume pairs should be equal; but in practice, small differences exist due to factors like differences in image artifacts and differences in sac isolations relative to the parent vessel. Thus there can be four definitions of aneurysm sac growth:

$$V_{SG1}=V_{S2+}-V_{S1-}$$ (1)

$$V_{SG2}=V_{S2+}-V_{S1+}$$ (2)

$$V_{SG3}=V_{S2-}-V_{S1-}$$ (3)

$$V_{SG4}=V_{S2-}-V_{S1+}$$ (4)

Further, in this retrospective study, scans for some time points—especially baseline scans—were not recorded and so some sac and coil volumes could not be estimated. Therefore, based on image artifact prevalence and scan availability, an additional sac growth definition (*V*_SG_) was prioritized among the above four definitions (or Eqs 1–4) in the following order: *V*_SG1_, *V*_SG2_, *V*_SG3_, *V*_SG4_. That is, if *V*_SG1_ was available then it was used as *V*_SG_, but if it was unavailable then *V*_SG2_ was used and so on. It should be noted that since the 2+ time point does not exist for control subjects, *V*_S2+_ is unavailable and consequently, *V*_SG1_ and *V*_SG2_ are incalculable for control subjects. Percent sac growth, % *V*_SG_, also followed a similar prioritized definition, where % *V*_SG_ = %*V*_SG1_ = *V*_SG1_/*V*_S1-_ if *V*_SG1_ was available, otherwise *V*_SG2_ was used and so on. To assess if use of a single *V*_SG_ definition (Eqs 1–4) led to a different interpretation of longitudinal aneurysm stability in this population, post-hoc statistical analyses (Paired Wilcoxon tests, p<0.05, one-tailed) using all of the data available for each definition were also done.

Volumetric coil mass growth, *V*_CG_, was computed as the difference in the coil mass volume between the 2- and the 1+ presentation:

$$V_{CG}=V_{C2-}-V_{C1+}$$ (5)

Unlike for sac growth, there is only a single definition for *V*_CG_. Using this definition *V*_CG_ < 0 indicates compaction of the coil mass. Additionally percent coil growth was defined as %*V*_CG_ = *V*_CG_/*V*_C1+_. Finally, the centers of the 3D coil mass models at initial (1+) and follow-up (2-) were identified by numerical integration. The numerical integration was verified using simple geometries. The translation of the coil mass center (δ) was defined as the distance between the coil mass centers at initial and follow-up time points.

To document the sensitivity of the image processing pipeline to the investigator performing the analysis a second user, blinded from the first, used it to calculate the aneurysm sac and coil mass volumes for a selected group of eight recurrence patients. The volumes computed by each data analysis investigator were compared.

### Statistical Analysis

Paired Wilcoxon tests (statistical significance at p<0.05, one-tailed) were conducted to independently assess whether the sac and coil mass volumes were different at follow-up—specifically to test the hypotheses that *V*_SG_ > 0 and *V*_CG_ < 0 for the recurrence and control cohorts. A Mann Whitney U-Test (p<0.05, one-tailed) was used to assess for differences in the translation of coil mass centers (δ) between recurrence and control cohorts.

## Results

**Fig 2:**
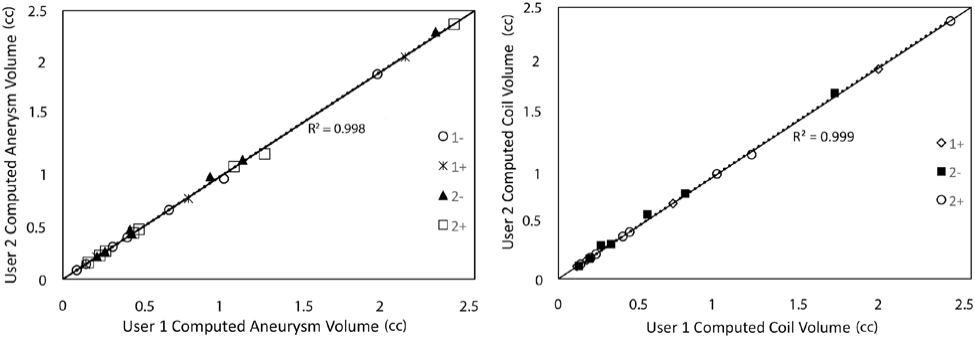
Comparison of the computed sac and coil volumes by two investigators blinded from each other’s results.

**Fig 3:**
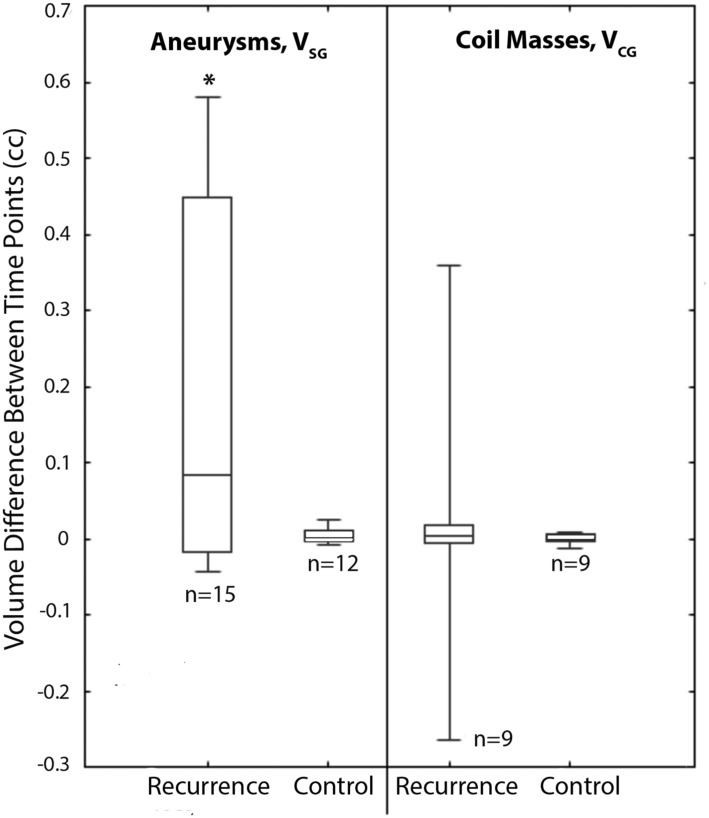
Aneurysm sac growth (*V*_SG_) and Coil mass growth (*V*_CG_) in the recurrence and control cohorts. The box and whisker plots show quartiles and the p-values are from paired one-tail Wilcoxon tests for hypothesizing that the aneurysm sac will grow (null hypothesis, *H*_0_: *V*_**SG**_ ≤ 0; alternative hypothesis, *H*_**A**_: *V*_**SG**_ > 0) and the coil mass will compact (*H*_0_: *V*_**CG**_ ≥ 0; *H*_**A**_: *V*_**CG**_ < 0).

**Fig 4:**
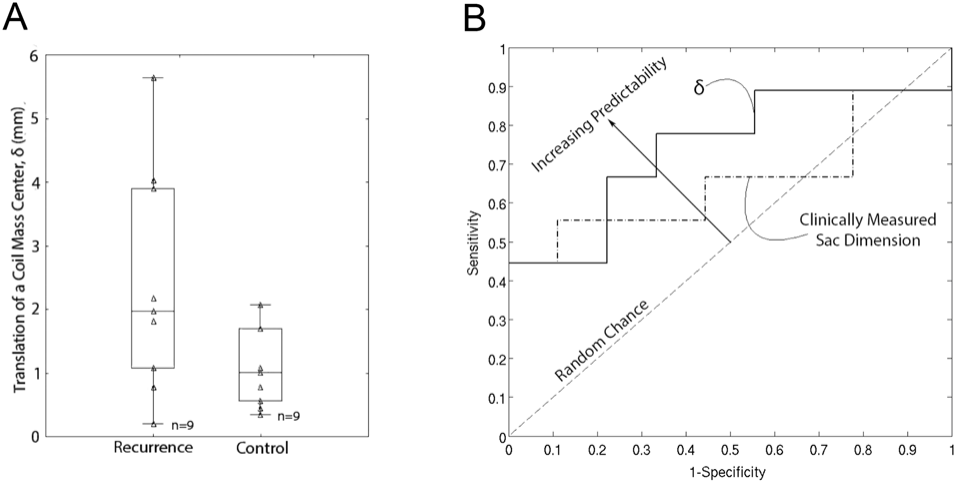
**(A) Coil mass center translation, δ, in the recurrence (N = 9) and control cohorts (N = 9)**. The box and whisker plots show quartiles and the p-values are from one-tail Mann-Whitney U test for hypothesizing that δ will be higher in the recurrence cohort than in control (null hypothesis, *H*_0_: δ_**RECR**_ ≤ δ_**CTRL**_; alternative hypothesis, *H*_**A**_: δ_**RECR**_ > δ_**CTRL**_). The triangles indicate raw data values. **(B) Receiver operator curve showing the predictability of δ in recurrence (N = 9) and control (N = 9) aneurysms.** δ is a better predictor of recurrence than clinically measured sac size, as it has a larger area under the curve or AUC (AUC = 0.74). Optimal sensitivity and specificity in differentiating recurrence aneurysms from control was found at δ = 1.1 mm.

Demographic and procedural study subject information is given in Table 1. The aneurysm sac and coil mass volumes estimated by the two blinded users agreed quite strongly (see Fig 2) with coefficients of determination for the aneurysm sac and coil mass volumes of 0.998 and 0.999 respectively. Imaging data was available to calculate *V*_SG_ in all 15 subjects within the recurrence cohort (11 from *V*_SG1_, 1 from *V*_SG2_, 2 from *V*_SG3_, and 1 from *V*_SG4_) and all 12 subjects in the control cohort (all 12 from *V*_SG3_). However imaging data was only available to calculate *V*_CG_ in 9 of 15 aneurysms in the recurrence cohort and 9 of 12 aneurysms in the control cohort. Aneurysm sac growth was found to exist with statistical significance in the recurrence cohort (min, median, max *V*_SG_ = -0.044, +0.083, +0.580 cc; p = 0.003; see Fig 3). In contrast, sac growth was not found to exist with statistical significance in the control cohort (min, median, max *V*_SG_ = -0.009, +0.001, +0.024 cc; p = 0.136; see Fig 3). Coil mass compaction was not found to occur with statistical significance in either the recurrence (min, median, max *V*_CG_ = -0.265, +0.004, +0.359 cc; p = 0.339; see Fig 3) or control cohorts (min, median, max *V*_CG_ = -0.013, 0.000, +0.008 cc; p = 0.429; see Fig 3). Translation of a coil mass center (δ) in the recurrence cohort was significantly larger than in the control cohort (min, median, max δ = 0.21, 1.97, 5.64 mm versus 0.32, 0.99, 2.06 mm; p = 0.047). It is noteworthy that the control cohort had smaller aneurysms at initial presentation compared to the recurrence cohort (sac size median = 10 versus 6.5 mm; 2p = 0.004 by Mann-Whitney U-test). A receiver operating characteristic (ROC) curve was plotted for δ and was found to have an area under the curve (AUC) of 0.74. Therefore δ is a better predictor of recurrence than clinically measured sac size, which had an AUC = 0.65 (see Fig 4). Sac size (height x width) of all aneurysms was measured by a single neurosurgical specialist on angiograms, the largest dimension of which was labeled as clinically measured sac size. According to the ROC curve, δ = 1.1 mm had optimal sensitivity and specificity to distinguish recurrence from control aneurysms.

**Table 1:** Study subject procedural and demographic information

Subject*	Age at Initial Presentation (Y)	SEX	Aneurysm Location*	Aneurysm Size (mm)	Ruptured Status	Follow-up Interval (Mo)
**01 (RE)**	72	F	L paraophthalmic	26x17	Unruptured	10.5
**02 (RE)**	58	F	L MCA	16x11	Unruptured	5.8
**03 (RE)**	51	F	L vertebral	14x11	Unruptured	6.7
**04 (RE)**	50	M	Acom	4x13	Unruptured	18.6
**05 (RE)**	53	F	Acom	10x8	Unruptured	24.3
**06 (RE)**	60	F	R pcom	9x8	Unruptured	16.8
**07 (RE)**	46	F	L cavernous ICA	7x8	Unruptured	6.6
**08 (RE)**	49	F	Basilar tip	6x6	Unruptured	5.9
**09 (RE)**	47	M	Basilar tip	22x15	Ruptured	5.3
**10 (RE)**	59	F	R pcom	16x11	Ruptured	11.5
**11 (RE)**	22	M	R MCA terminus	7x10	Ruptured	4.3
**12 (RE)**	58	M	Acom	5x10	Ruptured	5.7
**13 (RE)**	68	F	Acom	9x8	Ruptured	12.1
**14 (RE)**	61	F	Basilar tip	6x6	Ruptured	2.0
**15 (RE)**	52	F	Acom	4x4	Ruptured	5.8
**16 (C)**	37	F	L ophthalmic	9x9	Unruptured	5.4
**17 (C)**	50	F	L paraophthalmic	9x8	Unruptured	5.5
**18 (C)**	50	F	L cavernous ICA	8x8	Unruptured	5.5
**19 (C)**	69	F	R ICA	7x7	Unruptured	19.7
**20 (C)**	40	F	R paraophthalmic	7x7	Unruptured	6.5
**21 (C)**	16	F	R cavernous ICA	5x3	Unruptured	6.4
**22 (C)**	47	F	R ICA terminus	4x4	Unruptured	22.6
**23 (C)**	69	F	R ICA	2x3	Unruptured	19.7
**24 (C)**	65	F	R pcom	8x3	Ruptured	8.2
**25 (C)**	61	F	R pcom	6x5	Ruptured	6.3
**26 (C)**	45	F	Basilar tip	5x4	Ruptured	14.7
**27 (C)**	45	F	Acom	3x5	Ruptured	6.7

*RE indicates recurrence; C, control; F, female; M, male; L, left; R, right; MCA, middle cerebral artery; Acom, anterior communicating artery; Pcom, posterior communicating artery; ICA, internal carotid artery

## Discussion

We have developed a new, rigorous, and objective image analysis protocol to estimate aneurysm and coil mass volume growth. Additionally we computed the mass center of each coil mass in the study to assess whether the translation of the coil mass center serves as a marker for recurrence. Unlike previous studies, the aneurysm population in this study was free of any selection bias as it included all the aneurysms that presented with recurrence at the UIHC between July 2009 and September 2012 for which 3DRA scans were available. Further, this is the first study to include control aneurysms (consecutively chosen). Study of the non-recurrence control subjects serves the explicit purpose of revealing any inherent bias in the image analysis methodologies that could predispose the calculations to identifying growth. Additionally the automated image analysis protocol was shown to be objective and insensitive to the investigator (see Fig 2). Finally, this is the first study to assess if the translation of the coil mass center is measurably greater in coil embolized aneurysms that present with recurrence.

Aneurysm sac growth was found to be associated with recurrence in this study with sensitivity (since sac growth was noted in the recurrence cohort) and specificity (since sac growth was not noted in the control cohort, see Table 2). Coil mass compaction was not found to be associated with recurrence since it was not noted with enough consistency in either the recurrence or control cohorts (see Table 2). These findings are consistent with De Crane et al.’s earlier report[10] based on 3D image processing methods and also with a very recent report by Abdihalim et al.[16] that is based on 2D manual measurements; Abdihalim et al.[16] found 11 of 29 aneurysms had coil compaction while 18 of 29 exhibited sac growth.

**Table 2:** Outcome summary of the paired one-tailed Wilcoxon tests for the recurrence and control cohorts.

Cohort	Definition of Growth	mean growth ± SD of paired differences (cc)	mean %growth ± SD of paired differences (cc)	p value	n
**Recurrence**	V_SG_	0.194 ± 0.228	26 ± 38	0.003	15
	V_SG1_	0.229 ± 0.227	39 ± 33	0.003	11
	V_SG2_	0.203 ± 0.190	31 ± 28	0.023	6
	V_SG3_	0.183 ± 0.214	23 ± 31	0.011	12
	V_SG4_	0.124 ± 0.144	21 ± 25	0.013	8
	V_CG_	0.010 ± 0.164	7 ± 19	0.339	9
**Control**	V_SG1_	N/A	N/A	N/A	N/A
	V_SG2_	N/A	N/A	N/A	N/A
	V_SG3_	0.005 ± 0.011	1 ± 14	0.136	12
	V_SG4_	-0.012 ± 0.037	-6 ± 26	0.384	9
	V_CG_	0.00007 ± 0.006	6 ± 23	0.429	9

Overall, sac growth requires far greater attention than is currently accorded to it. If these findings are taken at face value, it has some important implications. For example, perhaps a growing aneurysm or one deemed to be at a high risk of growth is also at a high risk for recurrence and hence better treated by other modalities. Finally, if coil compaction is rare, it also has implications for coil design. An interesting observation in this study was that the coil masses in nine aneurysms grew during follow-up (these nine were from both the recurrence and control cohorts). Incidentally, we found that 8 of the 9 were coiled using hydrocoils. The likely explanation for the coil mass growth in the one aneurysm not coiled with hydrocoils is that image artifacts present in the 1+ baseline 3DRA scan led to an underestimated 1+ coil mass volume. Because this aneurysm was also one of the smallest aneurysms analyzed for coil mass compaction (0.064 cc sac volume at initial presentation) such an underestimation was enough to result in an indication of longitudinal coil mass growth. Finding that hydrocoils were used in all but one aneurysm with indicated coil mass growth adds credibility to our methods, yet also shows that image artifacts associated with the metal coil mass cannot be discounted in the methodology.

Additionally the findings of this study are consistent with the hypothesis that the translation of coil mass center is greater in aneurysms that present with recurrence. This hypothesis arises from the idea that sac growth would merely cause the coil mass to shift deeper into the sac to permit recanalization at the neck and has implications for management of patients treated by coil embolization. Currently coil embolization patients are longitudinally followed by angiographic scans, which are costly, inconvenient and pose some risks. If the hypothesis is valid, coil mass center translation can serve as an image biomarker for recurrence and importantly, since the coil mass is visible on bone scans, non-angiographic scanning may itself suffice for monitoring these patients. Of course, all this rests on the sensitivity and specificity of δ as an image biomarker. In this context, the fact that the control cohort was smaller than the recurrence cohort is a key limitation to consider even though coil mass translation was found to have better sensitivity and specificity compared to clinically measured sac size. The potential for coil mass translation to serve as an image biomarker needs to be evaluated in larger and size matched study populations before it can serve as a reliable biomarker for recurrence.

Some limitations of the study design are worth considering, for instance this is a single-center, single-practitioner experience. The sample size, although largest of its nature to date, is limited and the recurrence cohort subjects in this study include those from our pilot study[9] reported earlier. Despite the significant advances introduced in the analysis protocol in this paper, image artifacts, especially blind spots—or regions where a clot exists without coils and hence blind to both the bone and subtraction 3DRA scans—are a cause for some concern. Visual observations of gaps in the sac surface of some 3D models, especially those corresponding to the 2- time point, suggest the possibility of such blind spots. Fortunately since there was a redundancy of image data for sac growth calculations we could assess their effect. In post-hoc analyses, we repeated the statistical tests by defining sac growth as Eqs 1–4. Statistically significant sac growth was found in the recurrence cohort regardless of the growth definition used (p values of 0.003, 0.023, 0.011, and 0.013) but not in the control cohort (p values of N/A, N/A, 0.136 and 0.384; note that *V*_SG1_ and *V*_SG2_ do not exist for control aneurysms because they do not have 2+ time point, see Table 2). Therefore, our definition for *V*_SG_, while expedient, has little impact on the findings. Finally the image analysis protocol, though highly automated, does require some investigator interaction; however, the sensitivity study clearly demonstrated that the impact from such investigator-made choices is negligible (see Fig 2). It has to be noted that all these tests only establish consistency in our methods, not necessarily accuracy because we lack a ground truth to compare to. Nevertheless finding no evidence of sac growth in the control cohort suggests there is no bias in the computations performed and sac growth is indeed associated with sensitivity and specificity to recurrence.

In this context it is worth noting that the control cohort was smaller than the recurrence cohort. Specifically 9 of the 15 aneurysms in the recurrence cohort were clinically measured to be 10 mm in size or larger; in contrast, no aneurysm in the control cohort was this large. Furthermore both cohorts contained 8 unruptured aneurysms while the remaining 7 recurrence aneurysms and 4 control aneurysms were ruptured. Nevertheless we do not believe these differences in cohort characteristics adversely impact the findings made. This is because the aneurysms chosen for each cohort were done in an inclusive and consecutive manner to avoid selection bias; furthermore, the recurrence cohort consists of a fairly proportionate number of large to small aneurysms and unruptured to ruptured aneurysms. Finding no evidence of coil compaction in such a cohort seems to indicate that coil compaction is not associated with recurrence regardless of aneurysm size or rupture status.

## Conclusion

In our study we found there was no association between recurrence in coil embolized cerebral aneurysms and coil compaction; particularly, we found aneurysm sac growth to be the predominant etiology of recurrence. In addition our findings support the hypothesis that translation of the coil mass within the aneurysm sac has potential to serve as a marker for recurrence if verified independently with a size-matched control cohort. This particular finding suggests that there is potential for non-angiographic imaging to diagnose cerebral aneurysm recurrence with sensitivity and specificity.
